# Enhanced Antimalarial Efficacy of *Annona muricata* Leaf Extract Combined With Artesunate, Chloroquine, and Pyrimethamine in *Plasmodium berghei*–Infected ICR Mice

**DOI:** 10.1155/adpp/8736555

**Published:** 2025-08-07

**Authors:** Orawan Sarakul, Rachasak Boonhok, Voravuth Somsak

**Affiliations:** ^1^School of Allied Health Sciences, Walailak University, Nakhon Si Thammarat 80160, Thailand; ^2^Hematology and Transfusion Science Research Center, Walailak University, Nakhon Si Thammarat 80160, Thailand; ^3^Research Excellence Center for Innovation and Health Products (RECIHP), Walailak University, Nakhon Si Thammarat 80160, Thailand

## Abstract

Malaria continues to be a significant global health challenge, particularly in tropical and subtropical regions, due to its high morbidity and mortality rates. The development of resistance to conventional antimalarial drugs underscores the urgent need for novel therapeutic approaches. Artemisinin-based combination therapies (ACTs) are effective but face emerging resistance issues. This study explores the antimalarial efficacy of *Annona muricata* leaf extract (AME) when combined with artesunate (ART), chloroquine (CQ), and pyrimethamine (PYR) in *Plasmodium berghei*–infected ICR mice. Fresh *A. muricata* leaves were processed to produce a crude ethanolic extract. ART, CQ, and PYR were prepared and administered to ICR mice infected with *P. berghei* ANKA. The study evaluated the parasitemia levels and survival rates, comparing combination treatments to monotherapies. The combination treatments were analyzed for synergistic interactions. Results indicated that AME alone exhibited significant antimalarial activity, especially at higher doses. The combination of AME with ART and PYR demonstrated significant synergistic effects, achieving over 90% inhibition of parasitemia and significantly prolonging mean survival times up to 30 days. However, the combination of AME with CQ did not show synergistic effects. These findings suggest that AME, particularly in combination with ART or PYR, could enhance antimalarial efficacy and offer a promising alternative to current treatments, potentially mitigating drug resistance issues. Further research is warranted to validate these combinations and explore their mechanisms of action.

## 1. Introduction

Malaria remains a major public health challenge, particularly in tropical and subtropical regions, where it causes significant morbidity and mortality. Despite extensive efforts to control and eliminate malaria, the disease continues to claim hundreds of thousands of lives annually, primarily affecting children under the age of five and pregnant women [1]. The causative agent, *Plasmodium* spp., has developed resistance to most antimalarial drugs, including chloroquine (CQ) and pyrimethamine (PYR), which complicates treatment strategies and necessitates the development of novel therapeutic approaches [2, 3].

Artemisinin-based combination therapies (ACTs) are currently the cornerstone of malaria treatment due to their rapid action and efficacy against multidrug-resistant *Plasmodium* strains. However, emerging resistance to artemisinin and its derivatives poses a serious threat to malaria control programs worldwide [4, 5]. Consequently, there is an urgent need to explore alternative antimalarial agents, particularly those derived from natural sources, to augment existing treatment regimens.

*Annona muricata*, commonly known as soursop or graviola, is a tropical plant traditionally used in various cultures for its medicinal properties [6]. *A. muricata*'s activities were shown to include antioxidant, anti-inflammation, antihemolytic, anticancer, antiulcer, antidiabetic, antiprotozoa, antidiarrhea, antibacteria, antiviral, antihypertensive, and wound healing [7]. Recent studies have highlighted its potential antimalarial activity, attributed to bioactive compounds such as acetogenins, alkaloids, and flavonoids [7–10]. These compounds have shown promising results in inhibiting *Plasmodium falciparum* growth in vitro and reducing parasitemia in vivo [11–13]. Combination therapies are essential in combating drug resistance, as they can produce synergistic effects where the combined efficacy of the drugs exceeds the sum of their individual actions [14]. In the case of *A. muricata*, combining it with standard antimalarial drugs could not only enhance parasite clearance but also reduce the required dosages of conventional therapies. This could help mitigate potential side effects and slow the development of drug resistance [15]. The complementary mechanisms of action between *A. muricata* and antimalarial drugs further emphasize the importance of investigating these combinations, as they may offer a more effective and sustainable treatment strategy. Given the increasing resistance to conventional antimalarials and the potential efficacy of *A. muricata*, this study investigates the combined antimalarial effects of *A. muricata* leaf extract (AME) with artesunate (ART), CQ, and PYR. We hypothesize that the combination of AME with these standard antimalarials will exhibit synergistic effects, enhancing overall antimalarial efficacy and potentially mitigating the development of resistance.

To test this hypothesis, we conducted an experimental study using ICR mice infected with *Plasmodium berghei* ANKA. The primary objectives were to assess the parasitemia levels and survival rates in treated mice, compare the efficacy of combination treatments with monotherapies, and evaluate the potential synergistic interactions between AME and standard antimalarial drugs. This research aims to provide insights into alternative combination therapies that could improve malaria treatment outcomes and contribute to global efforts in combating this devastating disease.

## 2. Materials and Methods

### 2.1. Plant Material and Preparation of Extract

Fresh leaves of *Annona muricata* were collected from Royal Project Foundation, Chiang Mai, Thailand, and authenticated by Faculty of Pharmacy, Chiang Mai University. The leaves were washed, air-dried, and pulverized into a fine powder. The powdered leaves (200 g) were macerated in 1000 mL of 100% ethanol for 72 h with occasional shaking. The mixture was filtered using Whatman No. 1 filter paper, and the filtrate was concentrated under reduced pressure using a rotary evaporator at 40°C to yield a crude ethanolic extract. The extract (AME) was then dried to a constant weight in a desiccator and stored at 4°C until use.

### 2.2. Preparation of ART, CQ, and PYR

ART, CQ, and PYR were purchased from Sigma-Aldrich (St. Louis, MO, United States). ART powder was dissolved in a few drops of 1% sodium bicarbonate solution and then diluted with distilled water (DW) to achieve the chosen concentration. CQ powder at chosen doses was dissolved in DW. PYR powder was dissolved in a few drops of 70% ethanol and then diluted with DW to achieve the chosen concentration. Fresh solutions, presented in mg/kg of mouse body weight, were prepared daily and adjusted before administration by oral gavage according to the weight of the mice.

### 2.3. Animals

Male ICR mice, aged 6–8 weeks and weighing 20–25 g, were obtained from Nomura Siam International Co, Ltd., Bangkok, Thailand. All mice were housed in a pathogen-free environment with a 12-hour light/dark cycle and were provided with standard rodent chow and water *ad libitum*. For anesthesia, Zoletil 50 (a combination of tiletamine and zolazepam) was used. Mice were administered 30 mg/kg of Zoletil via intraperitoneal injection to induce general anesthesia. The depth of anesthesia was monitored by assessing the lack of response to gentle toe pinching and the absence of respiratory movement. Mice were kept under anesthesia for the duration of the experiment, ensuring minimal distress. Euthanasia was performed by administering an overdose of Zoletil 50 (100 mg/kg, intraperitoneal injection), following institutional guidelines for humane euthanasia. The procedure was confirmed by the absence of a heartbeat and reflexes. All experimental procedures were authorized by the Western University Animal Care and Use Committee (Approval No. WU2023-16) following the Code of Practice for the Care and Use of Animals for Scientific Purposes, established by the National Committee for Research Animal Development of the National Research Council of Thailand. All methods adhered to the relevant guidelines and regulations outlined in the National Research Council's Guide for the Care and Use of Laboratory Animals.

### 2.4. Parasite Strain

*Plasmodium berghei* ANKA strain (PbANKA) was obtained from the Malaria Research and Reference Reagent Resource Centre (MR4) and maintained by serial passage in ICR mice. The parasites were cryopreserved and thawed prior to infection of the experimental mice.

### 2.5. Investigation of Effective Dose

The standard 4-day chemosuppressive test was employed to determine the effective dose (ED_50_) of the drugs (AME, ART, CQ, and PYR) [16]. ICR mice (*n* = 5 per treatment group) were inoculated intraperitoneally with 6 × 10^6^ PbANKA-infected erythrocytes. Two hours postinfection, the mice received oral administration of AME (10, 50, 100, and 500 mg/kg), ART (0.1, 1, 5, 10, and 20 mg/kg), CQ (0.1, 1, 5, 10, 20 mg/kg), and PYR (0.1, 1, 5, 10, and 20 mg/kg) once daily for four consecutive days (D0–D3). The untreated group was given 10 mL/kg of DW to account for the administration volume. This vehicle control was included to ensure that any observed effects were due to the treatments rather than the administration process itself. On day 4, parasitemia was measured, and the percentage inhibition was calculated. The optimal ED_50_ value was determined through nonlinear regression to estimate the dose–response variable slope.

### 2.6. Combination Antimalarial Treatment

The ED_50_ values of AME, ART, CQ, and PYR were utilized to assess the combination treatment. The extract and drugs were combined in fixed ratio (1:1) of ED_50_, ED_50/2_, ED_50/4_, and ED_50/8_. This combination was tested using the standard 4-day chemosuppressive test [17]. Mice were infected with PbANKA and subsequently treated with AME, ART, CQ, or PYR, either alone or in combination once daily via oral gavage for four consecutive days, starting 2 hours after infection. The treatment regimen was continued daily through day 3, with the final parasitemia measurement taken on day 4, following the last dose of treatment. Parasitemia was measured and the percentage inhibition was calculated. Data points above the joint line indicated synergism, while those near or below the line indicated additive or antagonistic interactions, respectively. Additionally, a combination index (CI) value was performed using CompuSyn software as indicated in the Statistical Analysis section.

### 2.7. Parasitemia Assessment

Parasitemia was monitored at day 4. Blood samples were collected from the tail vein of each mouse on microscopic slides. Thin blood smears were prepared, fixed with 100% methanol, and stained with 10% Giemsa solution for 20 min. The smears were then washed with phosphate-buffered saline (PBS), air-dried, and analyzed under a light microscope at 1000x magnification. The number of infected erythrocytes was counted out of a total of 1000 erythrocytes, and parasitemia was calculated as the percentage of infected erythrocytes relative to the total number of erythrocytes [18].

(1) $$\begin{array}{c} \text{Parasitemia}\left(\%\right)=\left(\frac{\text{Number of infected erythrocytes}}{\text{Total number of erythrocytes}}\right)\times 100. \end{array}$$

### 2.8. Evaluation of Antimalarial Activity

The antimalarial activity of each treatment was evaluated by calculating the percentage inhibition in parasitemia compared to the untreated control. The percentage inhibition of parasitemia was calculated using the formula:

(2) $$\begin{array}{c} \text{Inhibition}\left(\%\right)=\left(\frac{\text{Parasitemia of untreated group}-\text{Parasitemia of tested group}}{\text{Parasitemia of untreated group}}\right)\times 100. \end{array}$$

### 2.9. Assessment of Mean Survival Time (MST)

To assess the efficacy of AME, ART, CQ, and PYR, both individually and in combination, in extending the survival of PbANKA-infected ICR mice, the MST was assessed [18]. Daily monitoring of mortality was conducted, and the duration from the time of infection until death was recorded for each mouse in both the treatment and control groups over a 30-day follow-up period. MST was then calculated using the following formula:

(3) $$\begin{array}{c} \text{MST}=\frac{\sum \text{Survival days of all mice in the group}}{\text{Number of mice in the group}}. \end{array}$$

### 2.10. Statistical Analysis

Data were expressed as mean ± standard error of mean (SEM). The optimal ED_50_ value was determined using nonlinear regression with a variable slope dose–response model. Statistical analysis was performed using one-way analysis of variance (ANOVA) followed by Tukey's post hoc test for multiple comparisons. Differences were considered statistically significant at *p* < 0.05. All analyses were performed using GraphPad Prism Version 10.1 (GraphPad Software Inc., San Diego, CA, USA). The CI value, indicating synergism (CI < 1), additive effect (CI = 1), or antagonism (CI > 1), was calculated using CompuSyn software (ComboSyn Inc., USA) [19].

## 3. Results

### 3.1. Antimalarial Activities of AME Against PbANKA-Infected ICR Mice

**Figure 1:**
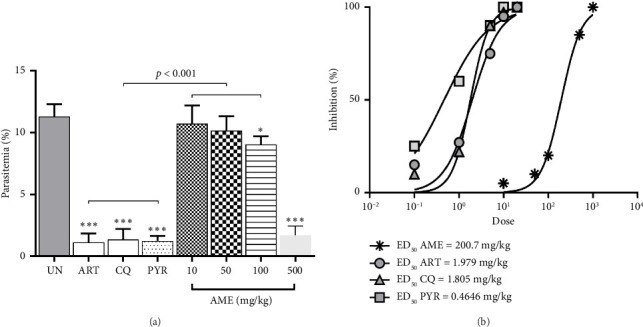
Antimalarial activity of AME against PbANKA-infected ICR mice. The mice were infected with PbANKA and then treated orally with AME (10, 50, 100, and 500 mg/kg) for four consecutive days. ART (10 mg/kg), CQ (10 mg/kg), and PYR (5 mg/kg) were used as positive controls. (a) Parasitemia levels were measured, and (b) effective doses of AME, ART, CQ, and PYR against PbANKA-infected ICR mice were determined. Results were presented as mean ± SEM (*n* = 5). ^∗^*p* < 0.05 and ^∗∗∗^*p* < 0.001 compared to UN. UN, untreated group; AME, *A. muricata* extract; ART, artesunate; CQ, chloroquine phosphate; PYR, pyrimethamine.

AME demonstrated significant (*p* < 0.05 and *p* < 0.001) antimalarial activity against PbANKA-infected ICR mice at doses of 100 mg/kg and 500 mg/kg, achieving 20% and 85% inhibition, respectively, compared to the untreated control (Figure 1(a)). However, doses of 10 mg/kg and 50 mg/kg did not show significant inhibition. Notably, AME at 500 mg/kg exhibited the highest antimalarial activity, comparable to 10 mg/kg of ART, 10 mg/kg of CQ, and 5 mg/kg of PYR. Additionally, the ED_50_ values derived from the dose–response curves were approximately 200 mg/kg for AME, 2 mg/kg for ART, 1.8 mg/kg for CQ, and 0.5 mg/kg for PYR (Figure 1(b)).

### 3.2. Combination Treatment of AME With ART, CQ, and PYR Against PbANKA-Infected ICR Mice

**Figure 2:**
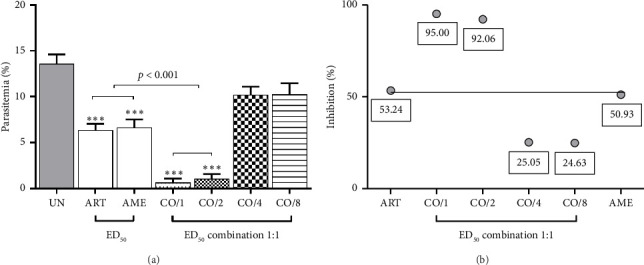
Combination treatment of ART and AME against PbANKA-infected ICR mice was evaluated. The mice were infected with PbANKA and then treated orally with a combination of ART and AME (1:1) at ED_50_, ED_50/2_, ED_50/4_, and ED_50/8_ doses for four consecutive days. (a) Parasitemia levels were measured, and (b) an interaction line was generated. Results were presented as mean ± SEM (*n* = 5). ^∗∗∗^*p* < 0.001 compared to the UN. UN, untreated group; ART, artesunate; AME, *A. muricata* extract; CO/1, CO/2, CO/4, and CO/8: combination treatment of ART and AME at ED_50_, ED_50/2_, ED_50/4_, and ED_50/8_ doses, respectively.

**Figure 3:**
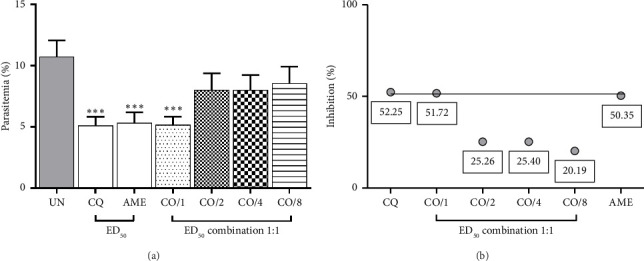
Combination treatment of CQ and AME against PbANKA-infected ICR mice was evaluated. The mice were infected with PbANKA and then treated orally with a combination of CQ and AME (1:1) at ED_50_, ED_50/2_, ED_50/4_, and ED_50/8_ doses for four consecutive days. (a) Parasitemia levels were measured, and (b) an interaction line was generated. Results were presented as mean ± SEM (*n* = 5). ^∗∗∗^*p* < 0.001 compared to the UN. UN, untreated group; CQ, chloroquine phosphate; AME, *A. muricata* extract; CO/1, CO/2, CO/4, and CO/8: combination treatment of ART and AME at ED_50_, ED_50/2_, ED_50/4_, and ED_50/8_ doses, respectively.

As shown in Figure 2, the combination of ART and AME at ED_50_ and ED_50/2_ doses exhibited significant (*p* < 0.001) antimalarial activity, achieving 95% and 92.06% inhibition, respectively, compared to the untreated control. Furthermore, the combination of ART and AME at these doses demonstrated a significantly (*p* < 0.001) more potent antimalarial effect than either ART or AME alone. The synergistic effect of this combination was indicated by a CI value of less than 1.0 (Table 1). However, combination of CQ and AME did not exhibit a synergistic effect, unlike the combination of ART and AME when treated individually (Figure 3 and Table 2).

**Figure 4:**
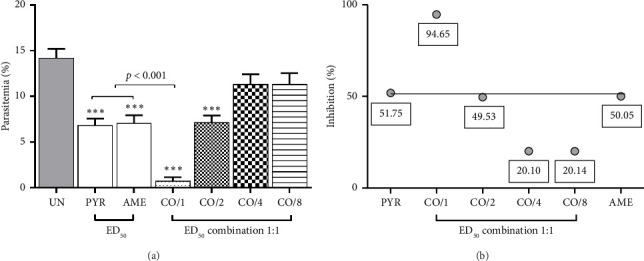
Combination treatment of PYR and AME against PbANKA-infected ICR mice was evaluated. The mice were infected with PbANKA and then treated orally with a combination of PYR and AME (1:1) at ED_50_, ED_50/2_, ED_50/4_, and ED_50/8_ doses for four consecutive days. (a) Parasitemia levels were measured, and (b) an interaction line was generated. Results were presented as mean ± SEM (*n* = 5). ^∗∗∗^*p* < 0.001 compared to the UN. UN, untreated group; PYR, pyrimethamine; AME, *A. muricata* extract; CO/1, CO/2, CO/4, and CO/8, combination treatment of ART and AME at ED_50_, ED_50/2_, ED_50/4_, and ED_50/8_ doses, respectively.

As shown in Figure 4, combination treatment of PYR and AME at ED_50_ demonstrated significant (*p* < 0.001) antimalarial activity compared to the untreated group with 94.65% inhibition. However, the combinations at ED_50/2_, ED_50/4_, and ED_50/8_ doses of PYR and AME displayed antagonistic interactions (Table 3).

### 3.3. Effect of Combination Treatment on MST in PbANKA-Infected ICR Mice

The MST is a key measure of the effectiveness of antimalarial treatments in extending the lifespan of infected mice. As demonstrated in Table 4, AME, ART, CQ, and PYR, when administered alone at their respective ED_50_ doses, significantly (*p* < 0.01) increased the MST compared to the untreated group. Notably, a significant (*p* < 0.001) prolongation of MST up to 30-day observation period was observed with the combination of AME and ART (at both ED_50_ and ED_50/2_ doses) and with AME and PYR (at ED_50_), highlighting their antimalarial efficacy. However, the combination of CQ and AME did not achieve a prolonged MST of up to 30 days.

## 4. Discussion

The present study evaluated the antimalarial efficacy of AME, ART, CQ, and PYR, both as monotherapies and in combination, against PbANKA-infected ICR mice. The MST results indicated that each treatment at the ED_50_ dose significantly prolonged the survival of infected mice compared to the untreated control group. This suggests a notable intrinsic antimalarial activity of the individual agents.

AME exhibited dose-dependent antimalarial effects, with significant inhibition observed at higher doses (100 mg/kg and 500 mg/kg). The highest antimalarial activity of AME at 500 mg/kg was comparable to that of standard antimalarial drugs ART, CQ, and PYR, indicating the potential of AME as a standalone antimalarial agent. These findings are consistent with previous research indicating the antimalarial properties of *A. muricata* [11]. The overall antimalarial efficacy of *A. muricata* might be attributed to its bioactive compounds, such as acetogenins, alkaloids, flavonoids, quinones, megastigmanes, and saponins, making this plant a valuable candidate in the quest for new antimalarial treatments [6, 7]. Specifically, acetogenins from *A. muricata* have shown potent cytotoxic properties against malaria parasites by disrupting the mitochondrial function of the parasite, resulting in its death [10, 20, 21]. This mechanistic understanding corroborates the observed efficacy of AME in the present study. Furthermore, the comparable efficacy of AME at 500 mg/kg to ART, CQ, and PYR is particularly noteworthy. These drugs are well-established antimalarial drugs with proven clinical efficacy. The fact that AME at 500 mg/kg can achieve similar antimalarial outcomes suggests that AME could be developed as a complementary or alternative treatment for malaria, particularly in areas where resistance to conventional drugs is prevalent. This is supported by findings from several reports, which indicated that natural extracts with potent antimalarial activity could serve as valuable alternatives in the management of drug-resistant malaria [22].

The combination therapy of AME with ART, particularly at ED_50_ and ED_50/2_ doses, showed a synergistic interaction, significantly enhancing antimalarial efficacy and extending MST up to the 30-day observation period. This synergism is confirmed by the CI values less than 1.0, demonstrating that combining AME with ART could reduce the required dose of ART while maintaining therapeutic efficacy. Such combinations could mitigate potential side effects and delay the development of resistance. The underlying mechanisms for this synergism may involve multiple factors. ART is known for its rapid action against malaria parasites, primarily by generating reactive oxygen species (ROS) that damage the parasite's cellular structures [23]. AME, on the other hand, contains acetogenins to disrupt mitochondrial function in the parasite, leading to cell death [21]. The combined action of these compounds with ART's mechanism can lead to enhanced parasite clearance. The results of this study align with previous findings that highlight the potential of plant-based compounds in combination therapies with ART. However, at lower doses, the plant extract could potentially interfere with the drug's mechanism or reduce its effectiveness, leading to antagonistic interactions.

Conversely, the combination of CQ and AME did not demonstrate a synergistic effect, and no significant extension in MST was observed. This lack of synergism suggests that CQ may not benefit from combination with AME, possibly due to pharmacodynamic or pharmacokinetic interactions that negate the expected enhancement in antimalarial activity. For instance, the bioactive compounds in AME may not enhance the absorption or bioavailability of CQ, or they may not complement the mechanism of action of CQ effectively, resulting in an additive or even antagonistic interaction. Additionally, while the 4-day chemosuppressive test utilized in this study is a standard approach for evaluating early-stage antimalarial efficacy, the decision to record parasitemia levels only on the fourth day may limit our ability to comprehensively assess the treatment's full impact. Parasitemia dynamics can vary over time, and monitoring parasitemia at a single time point does not provide insights into the progression of the infection or the temporal effects of treatment on parasitemia levels. Interestingly, the combination of PYR and AME at ED_50_ showed significant antimalarial activity with 94.65% inhibition. The possible mechanisms underlying this synergistic interaction can be attributed to the complementary actions of PYR and the bioactive compounds present in AME. PYR's inhibition of DHFR disrupts folate metabolism [24], while the bioactive compounds in AME exert cytotoxic effects through mitochondrial disruption and oxidative stress [25]. This multifaceted attack on the parasite can lead to a more effective reduction in parasitemia and a prolonged MST, as observed in the study. However, at lower doses (ED_50/2_, ED_50/4_, and ED_50/8_), antagonistic interactions may occur due to a suboptimal concentration of the plant extract, which might not provide the necessary therapeutic effects, or potentially alter the pharmacokinetics of the drug, reducing its overall efficacy. Additionally, at these lower doses, the plant extract might not sufficiently synergize with the drug, leading to diminished therapeutic activity. This indicates that while PYR and AME together at certain doses can be highly effective, improper dosing ratios might lead to suboptimal outcomes or antagonism.

Overall, these findings underscore the potential of *A. muricata* as an adjunct therapy in malaria treatment, particularly in combination with ART. The enhanced efficacy and extended MST observed with AME and ART combinations offer a promising therapeutic strategy. Moreover, the combination of AME with ART may allow for a reduction in the dose of ART required to achieve therapeutic efficacy. This dose reduction can be beneficial in several ways. Firstly, it can mitigate the potential side effects associated with ART, making the treatment more tolerable for patients. Secondly, by using lower doses of ART, the combination therapy can help delay the development of drug resistance, a significant concern in malaria treatment. Resistance to ART and other antimalarial drugs poses a major challenge in malaria control, and strategies that can extend the useful life of these drugs are highly valuable.

While this study highlights the antimalarial potential of AME in combination with ART, CQ, and PYR, several limitations should be considered. The study focused on early-stage parasitemia and did not assess long-term effects such as relapse or recrudescence. Future studies should include extended follow-up periods to evaluate the durability of treatment effects. Additionally, the study used a limited number of ICR mice infected with PbANKA. A larger sample size and multiple animal models could provide more robust and generalizable data. While various drug–extract combinations were tested, further dose–response analysis is needed to optimize the therapeutic ratios. Despite these limitations, the study suggests that AME could be a promising complementary treatment in malaria therapy, and future research should address these gaps to further investigate its therapeutic potential.

## 5. Conclusion

This study demonstrates the potential of AME as a viable adjunct to conventional antimalarial therapies. The combination of AME with ART and PYR significantly enhanced antimalarial efficacy in PbANKA-infected ICR mice, showing substantial reductions in parasitemia levels and extended survival times. These findings underscore the potential of AME to combat emerging resistance in malaria treatment, offering a promising natural alternative to current antimalarial drugs. While AME exhibited strong antimalarial activity on its own, its synergistic effects with ART and PYR highlight the importance of exploring natural compounds in combination therapies. The lack of synergy with CQ indicates the necessity for selective combination strategies to maximize therapeutic outcomes.

Overall, this research supports the integration of AME into malaria treatment regimens, particularly in regions burdened by drug-resistant *Plasmodium* strains. Future studies should focus on elucidating the mechanisms behind these synergistic interactions and conducting clinical trials to confirm the efficacy and safety of these combinations in humans.

In future studies, it will be crucial to extend the follow-up period to assess the long-term effects of AME on malaria parasite eradication and to determine whether the treatment prevents reinfection. Monitoring for relapse or recrudescence is particularly important in malaria studies, as the ability of a treatment to completely eliminate the parasite and prevent reinfection over time is essential to its classification as a curative therapy. Further studies should include additional endpoints such as the assessment of survival rates over extended periods, posttreatment parasitemia monitoring, and molecular analysis to detect any parasite recrudescence or resistance development. These measures will provide a more comprehensive understanding of AME's potential as a curative antimalarial agent. Moreover, the inclusion of a larger sample size and various parasite strains would help to validate the findings and confirm the broad-spectrum efficacy of AME.

## Figures and Tables

**Table 1:** Combination index of AME combined with ART against PbANKA-infected ICR mice.

Antimalarial test		Dose (mg/kg)		CI value
		ART	AME	
Combination (1:1)	ED_50_	2	200	0.65492^a^
	ED_50/2_	1	100	0.35919^a^
	ED_50/4_	0.5	50	1.72265^b^
	ED_50/8_	0.25	25	1.36132^b^

^a^CI < 1, synergism.

^b^CI > 1, antagonism.

**Table 2:** Combination index of AME combined with CQ against PbANKA-infected ICR mice.

Antimalarial test		Dose (mg/kg)		CI value
		CQ	AME	
Combination (1:1)	ED_50_	1.8	200	1.26514^a^
	ED_50/2_	0.9	100	1.32557^a^
	ED_50/4_	0.45	50	1.66278^a^
	ED_50/8_	0.225	25	1.83139^a^

^a^CI > 1, antagonism.

**Table 3:** Combination index of AME combined with PYR against PbANKA-infected ICR mice.

Antimalarial test		Dose (mg/kg)		CI value
		PYR	AME	
Combination (1:1)	ED_50_	0.5	200	0.51764^a^
	ED_50/2_	0.25	100	1.31044^b^
	ED_50/4_	0.125	50	1.39960^b^
	ED_50/8_	0.0625	25	1.39980^b^

^a^CI < 1, synergism.

^b^CI > 1, antagonism.

**Table 4:** MST of AME, ART, CQ, PYR, and its combination against PbANKA-infected ICR mice.

Antimalarial test		Dose (mg/kg)	MST (day)
Untreated group	—	10 mL/kg	8.00 ± 1.58

AME	ED_50_	200	20.80 ± 2.77^∗∗^
ART		2	27.00 ± 1.58^∗∗∗^
CQ		1.8	22.80 ± 3.11^∗∗^
PYR		0.5	23.40 ± 2.41^∗∗^

ART + AME	ED_50_	2 + 200	30.00 ± 0.00^∗∗∗^
	ED_50/2_	1 + 100	30.00 ± 0.00^∗∗∗^
	ED_50/4_	0.5 + 50	8.00 ± 1.32
	ED_50/8_	0.25 + 25	7.20 ± 1.92

CQ + AME	ED_50_	1.8 + 200	25.60 ± 2.30^∗∗^
	ED_50/2_	0.9 + 100	8.40 ± 1.21
	ED_50/4_	0.45 + 50	7.30 ± 1.82
	ED_50/8_	0.225 + 25	8.50 ± 1.67

PYR + AME	ED_50_	0.5 + 200	30.00 ± 0.00^∗∗∗^
	ED_50/2_	0.25 + 100	23.20 ± 1.34^∗∗^
	ED_50/4_	0.125 + 50	8.30 ± 1.62
	ED_50/8_	0.0625 + 25	8.40 ± 1.14

*Note:* ^∗∗^ *p* < 0.01 and ^∗∗∗^*p* < 0.001, compared to untreated group.

## Data Availability

The data that support the findings of this study are openly available in Figshare at https://figshare.com/s/e89c3de708dbada9a69c, reference number 10.6084/m9.figshare.26076313.
